# Supplementary material for: Genome-wide screening in pluripotent cells identifies Mtf1 as a suppressor of mutant huntingtin toxicity
Source: Nat Commun. 2023 Jul 5;14:3962. doi: 10.1038/s41467-023-39552-9 (PMC10322923; doi:10.1038/s41467-023-39552-9)
Supplement: Supplementary file 1 — Supplementary Information [file 41467_2023_39552_MOESM1_ESM.pdf]

Supplementary Information

A genome-wide screening in pluripotent cells identifies *Mtf1* as a suppressor of mutant huntingtin toxicity

Giorgia Maria Ferlazzo[1,8,9], Anna Maria Gambetta[1,2,9], Sonia Amato[2,3,9], Noemi Cannizzaro[1], Silvia Angiolillo[1], Mattia Arboit[1], Linda Diamante[2], Elena Carbognin[2], Patrizia Romani[1], Federico La Torre[2], Elena Galimberti[4], Florian Pflug[4], Mirko Luoni[5], Serena Giannelli[5], Giuseppe Pepe[6], Luca Capocci[6], Alba Di Pardo[6], Paola Vanzani[1], Lucio Zennaro[1], Vania Broccoli[5,7], Martin Leeb[4], Enrico Moro[1], Vittorio Maglione[6] and Graziano Martello[2].

1 Department of Molecular Medicine, Medical School, University of Padua, 35131, Padua, Italy
2 Department of Biology, University of Padova, Via U. Bassi 58B, 35131, Padua, Italy
3 Department of Neuroscience, University of Padova, Via Belzoni, 160, 35131, Padua, Italy
4 Max Perutz Laboratories Vienna, University of Vienna, Vienna Biocenter, Dr Bohr Gasse 9, 1030 Vienna
5 Division of Neuroscience, San Raffaele Scientific Institute, 20132, Milan, Italy
6 IRCCS Neuromed, 86077, Pozzilli, Italy
7 CNR Institute of Neuroscience, 20854 Vedrano al Lambro, Italy
8 Current address: Aptuit (Verona) S.r.l., an Evotec Company, Campus Levi-Montalcini, 37135, Verona, Italy
9 These authors contributed equally

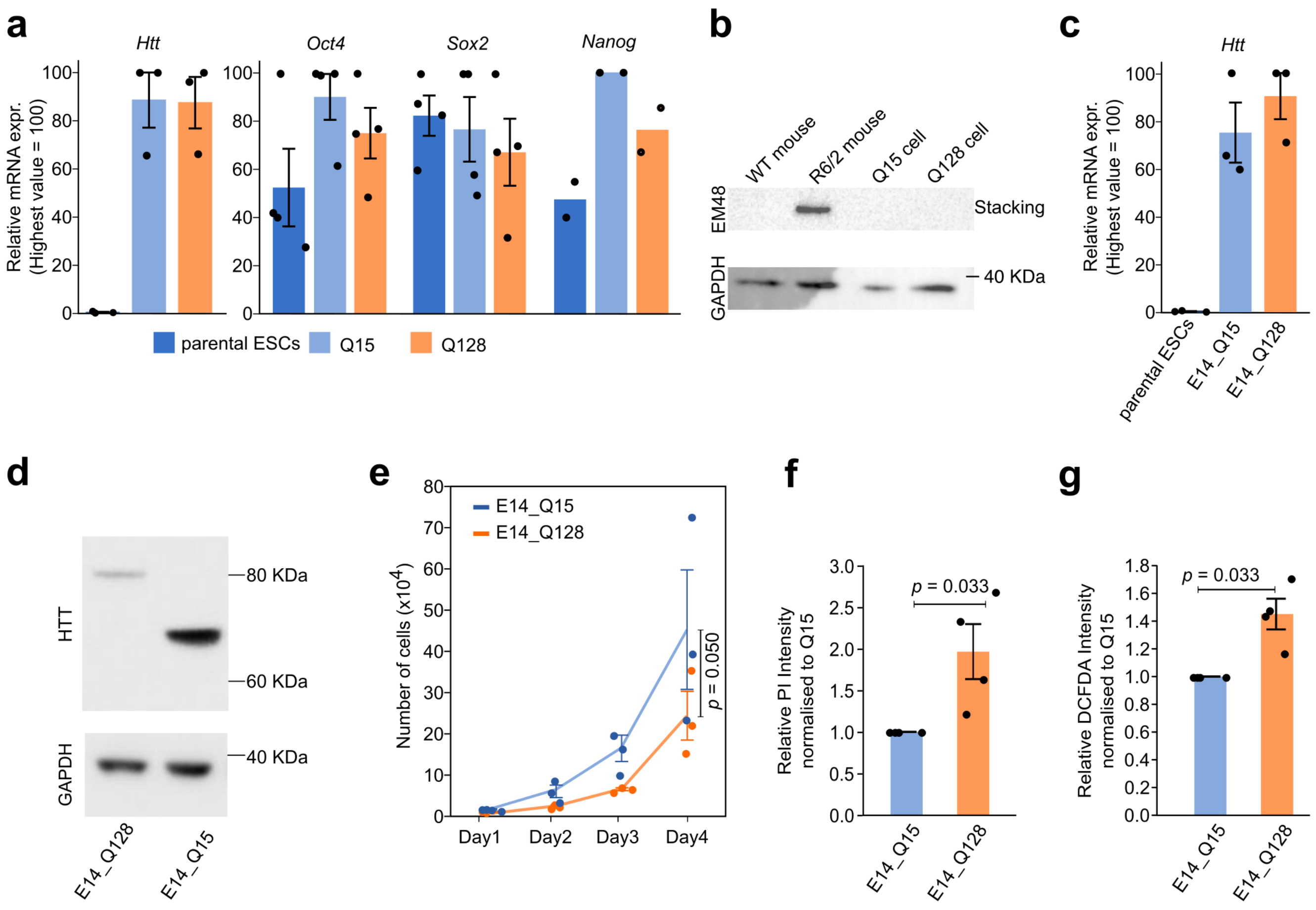
a
Htt
Oct4
Sox2
Nanog
Relative mRNA expr.
(Highest value = 100)
parental ESCs
Q15
Q128
b
WT mouse
R6/2 mouse
Q15 cell
Q128 cell
EM48
Stacking
GAPDH
40 KDa
c
Htt
Relative mRNA expr.
(Highest value = 100)
parental ESCs
E14_Q15
E14_Q128
d
HTT
80 KDa
60 KDa
GAPDH
40 KDa
E14_Q128
E14_Q15
e
E14_Q15
E14_Q128
Number of cells (x10^4)
p = 0.050
Day1
Day2
Day3
Day4
f
Relative PI Intensity
normalised to Q15
p = 0.033
E14_Q15
E14_Q128
g
Relative DCFDA Intensity
normalised to Q15
p = 0.033
E14_Q15
E14_Q128

Supplementary Figure 1 - Establishment and characterization of mHTT-expressing mouse ESCs

a Gene expression analysis by qPCR of *HTT* gene (on left) and pluripotency markers *Oct4, Sox2* and *Nanog* (right panels) in Q15 (blue) and Q128 (orange) cells, compared to parental mouse ESC line (Rex1GFP-d2). Bars indicate the mean ± SEM of 2 (for *Nanog*), 3 (for *HTT*) or 4 (for Poou5f1, also known as *Oct4,* and *Sox2*) independent experiments shown as dots. Expression was normalised to the highest value. **b** Western Blot for HTT aggregates (EM48 antibody) n=1 independent experiment. The stacking gel shows HTT aggregates only in R6/2 mouse brain lysate. HTT aggregates were absent in WT mouse brain lysate, Q15 and Q128 cells lysate. GAPDH was used as loading control. **c** Gene expression analysis by qPCR of *HTT* gene in WT mouse ESCs (E14IVc) expressing either Q15 (blue) or Q128 (orange) constructs, compared to parental mouse ESC line. Bars indicate the mean ± SEM of 3 independent experiments shown as dots. Expression was normalised to the highest value. **d** Western Blot of HTT confirmed the correct production of a 80 kDa and a 65 kDa form of HTT protein in E14_Q128 and E14_Q15 cells respectively. GAPDH was used as loading control. n=2 independent biological replicates. **e** Proliferation assay of E14_Q128 (orange) and E14_Q15 (blue) ESCs showed pronounced impairment in cell proliferation due to mHTT expression. Bars indicate the mean ± SEM of 3 independent experiments, shown as dots. *P*-values were calculated with Two-way Repeated Measure ANOVA. **f** Measurement of cell death by PI uptake followed by Flow Cytometry. E14_Q128 cells (orange) display higher cell death, compared to E14_Q15 (blue) cells. Bars indicate the mean ± SEM of 4 independent experiments shown as dots. FCs were calculated relative to the Q15 samples. *P*-values were calculated with One-tailed one sample Mann-Whitney U test. **g** Measurement of H_2DCFDA fluorescence as an evaluation of ROS production in E14_Q15 (blue) versus E14_Q128 (orange) cells. Bars indicate the mean ± SEM of four independent experiments shown as dots. FCs were calculated relative to the Q15 samples. *P*-values were calculated with One-tailed one sample Mann-Whitney U test. Uncropped gels and numerical values are provided in the Source data file.

a

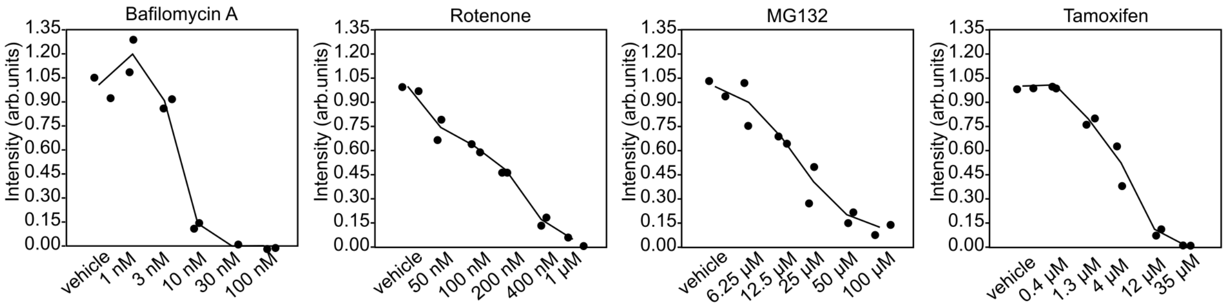

b

MG132 Clone	Gene Symbol	Description	Integration Site
7	Arnt2	Aryl-Hydrocarbon Receptor Nuclear Translocator 2	chr7:84,360,699-84,361,053
9	Chst5	Carbohydrate (N-Acetylglucosamine 6-O) Sulfotransferase 5	chr8:111,890,465-111,890,633
7	D930020B18Rik	RIKEN cDNA D930020B18 gene, mRNA	chr10:121,693,105-121,693,208
8	D930020B18Rik	RIKEN cDNA D930020B18 gene, mRNA	chr10:121,693,105-121,693,243
20	Epb41l3	Erythrocyte membrane protein band 4.1-like 3	chr17:69,192,378-69,192,625
21	Fbxo34	F-Box Protein 34	chr14:47,509,221-47,509,483
24	Fcer2	Low affinity immunoglobulin epsilon Fc receptor	chr8:3,685,125-3,685,209
15	Kdm5b	Lysine (K)-Specific Demethylase 5B	chr1:134,538,455-134,538,739
16	LOC102636514	LOC102636514, long non-coding RNA	chr8:57,315,708-57,315,834
18	Mtf1	Metal-Regulatory Transcription Factor 1	chr4:124,802,450-124,802,810
14	Npy5r	Neuropeptide Y Receptor Y5	chr8:66,630,962-66,989,630
3	Qkl	Quaking	chr17:10,258,394-10,258,593
5	Qkl	Quaking	chr17:10,258,394-10,258,570
6	Qkl	Quaking	chr17:10,258,636-10,259,069
12	Rab29	Member RAS Oncogene Family	chr1:131,820,182-131,886,459
10	Slc1a1	Solute Carrier Family 1, Member 1	chr19:28,732,773-28,733,099
9	Synj2	Synaptojanin 2	chr17:5,986,042-5,986,095
22	Trappc6b	Trafficking Protein Particle Complex 6B	chr12:59,080,863-59,080,886

Tamoxifen Clone	Gene Symbol	Description	Integration Site
4	Arid1b	AT Rich Interactive Domain 1B (SWI1-Like)	chr17:5,254,628-5,255,100
4	Fcer2	Low affinity immunoglobulin epsilon Fc receptor	chr8:3,685,125-3,685,283
7	Fcer2	Low affinity immunoglobulin epsilon Fc receptor	chr8:3,685,125-3,685,152
18	Fcer2	Low affinity immunoglobulin epsilon Fc receptor	chr8:3,685,125-3,685,191
8	Hdgf	Hepatoma-Derived (Heparin binding) Growth Factor	chr3:87,893,840-87,893,892
18	Mctp2	Multiple C2 Domains, Transmembrane 2	chr7:72,327,205-72,327,252
9	Pbld	Phenazine Biosynthesis-Like Protein Domain Containing	chr4:135,594,236-135,594,467
15	Plk3	Polo-Like Kinase 3	chr4:117,140,392-117,140,818
3	Qkl	Quaking	chr17:10,258,394-10,258,585
12	Qkl	Quaking	chr17:10,258,394-10,258,509
13	Qkl	Quaking	chr17:10,258,394-10,258,542
17	Scrt2	Scratch Family Zinc Finger 2)	chr2:152,051,425-152,051,474
6	Slc38a4	Solute Carrier Family 38, Member 4	chr15:97,097,874-97,097,994
7	Trak2	Trafficking Protein, Kinesin Binding 2	chr1:58,959,553-58,959,704
16	Wrn	Werner Syndrome, RecQ Helicase-Like	chr8:33,281,590-33,281,947

c **d** **e**

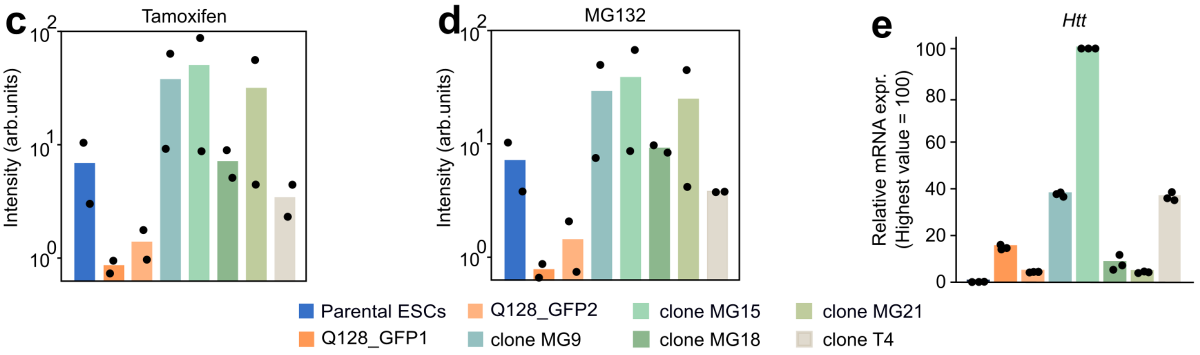

Supplementary Figure 2 - A gain-of-function screening for suppressors of mHTT toxicity

a Number of surviving parental mouse ESCs quantified by CV staining upon treatment for 48 hours with the indicated compounds. Lines indicate the mean of 2 independent experiments. Data were normalised to cells treated with vehicles. **b** List of target genes identified by Splinkerette-PCR and analysis of PCR bands for all MG or Tamoxifen clones collected. For 9 clones (out of 44 collected) we could not identify the site of integration for technical limitations, such as multiple integration sites or failure to obtain an amplicon. **c** CV quantification of surviving colonies in all 5 clones in analysis after 48 hours of treatments with Tamoxifen. Bars indicate the mean of 2 independent experiments shown as dots. **d** CV quantification showing the number of surviving colonies in all 5 clones in analysis after 48 hours of treatments with MG132. Bars indicate the mean of 2 independent experiments shown as dots. **e** qPCR analysis showed similarly high expression levels of *HTT* mRNA in all clones, as well as in Q128 cells expressing GFP, confirming that *mHTT* mRNA was not silenced during the screening procedure. Bars indicate the mean of 3 technical replicates shown as dots. Expression was normalised to the highest value. Numerical values are provided in the Source data file.

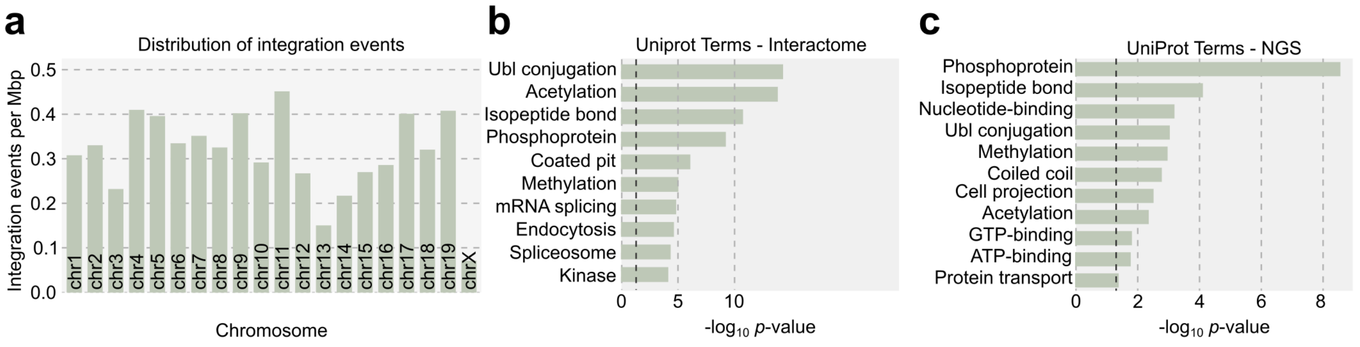
a
Distribution of integration events
Integration events per Mbp
0.0
0.1
0.2
0.3
0.4
0.5
chr1
chr2
chr3
chr4
chr5
chr6
chr7
chr8
chr9
chr10
chr11
chr12
chr13
chr14
chr15
chr16
chr17
chr18
chr19
chrX
Chromosome
b
Uniprot Terms - Interactome
Ubl conjugation
Acetylation
Isopeptide bond
Phosphoprotein
Coated pit
Methylation
mRNA splicing
Endocytosis
Spliceosome
Kinase
0
5
10
-log10 p-value
c
UniProt Terms - NGS
Phosphoprotein
Isopeptide bond
Nucleotide-binding
Ubl conjugation
Methylation
Coiled coil
Cell projection
Acetylation
GTP-binding
ATP-binding
Protein transport
0
2
4
6
8
-log10 p-value

Supplementary Figure 3 - Network analysis of candidate suppressors of mHTT toxicity

a Distribution of integration events normalised to the length of each chromosome in Megabases. **b** Gene list enrichment analysis for the genes represented in the interactome in Fig. 3b revealed a statistically significant enrichment for categories linked to proteasome degradation (e.g. ubiquitin conjugation, p-value = 5.7e-15) or vesicular trafficking (e.g. acetylation, p-value = 1.66e-14) and post-transcriptional regulation (e.g. methylation, p-value = 1.01e-5). P-values were calculated by two-tailed Fisher Exact test using the DAVID database. **c** Gene list enrichment analysis for all genes identified from NGS screenings, revealed a statistically significant enrichment for categories linked to proteasome degradation (e.g. ubiquitin conjugation, p-value = 9.77e-4) or vesicular trafficking (e.g. acetylation, p-value = 0.005) and transcriptional regulators (e.g. methylation, p-value = 0.001). P-values were calculated by two-tailed Fisher Exact test using the DAVID database.

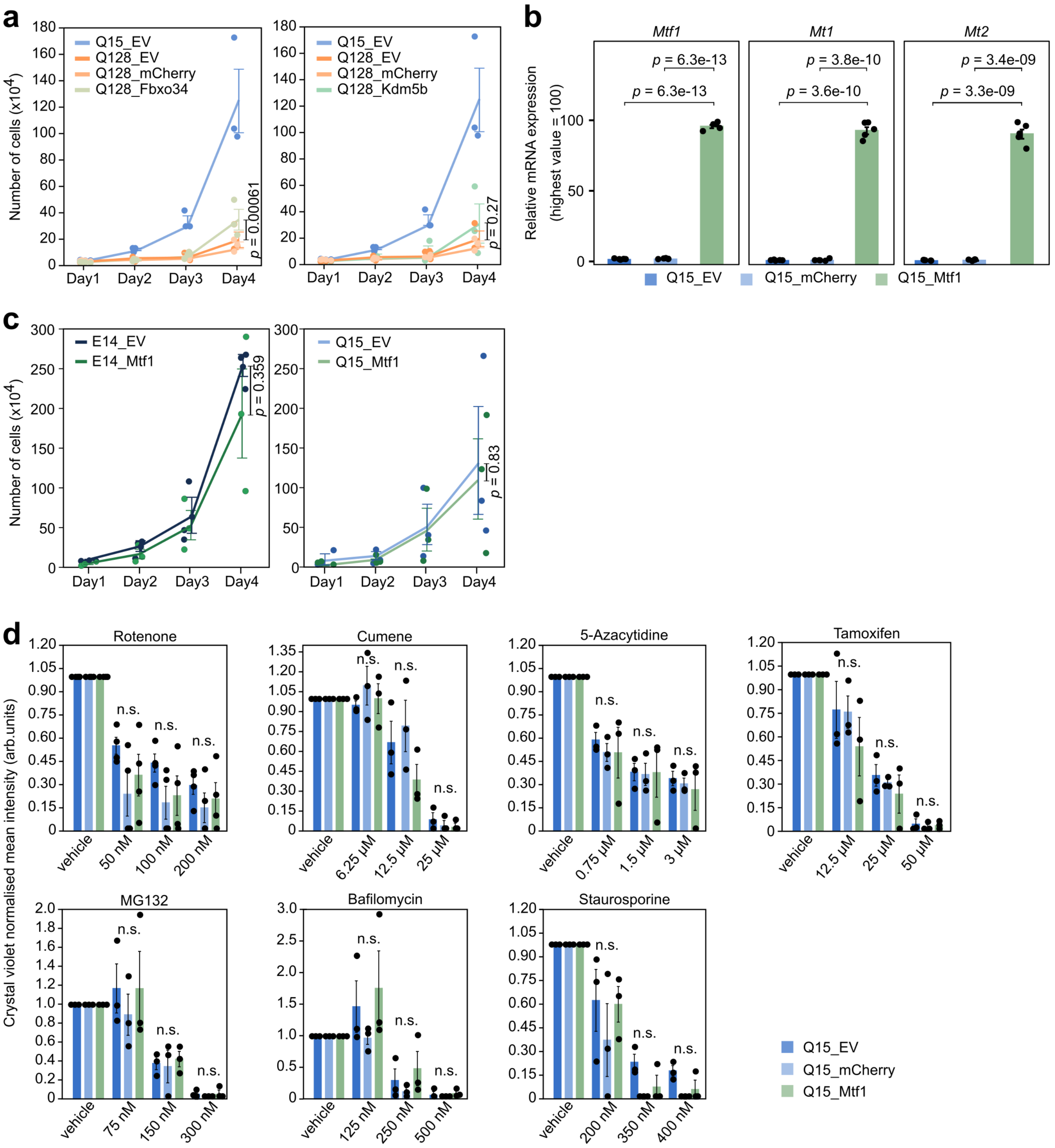
a
Number of cells (x10^4)
Q15_EV
Q128_EV
Q128_mCherry
Q128_Fbxo34
Q128_Kdm5b
p = 0.00061
p = 0.27
Day1
Day2
Day3
Day4
b
Mtf1
Mt1
Mt2
Relative mRNA expression (highest value = 100)
p = 6.3e-13
p = 6.3e-13
p = 3.8e-10
p = 3.6e-10
p = 3.4e-09
p = 3.3e-09
Q15_EV
Q15_mCherry
Q15_Mtf1
c
E14_EV
E14_Mtf1
Q15_EV
Q15_Mtf1
p = 0.359
p = 0.83
d
Crystal violet normalised mean intensity (arb.units)
Rotenone
vehicle
50 nM
100 nM
200 nM
Cumene
6.25 μM
12.5 μM
25 μM
5-Azacytidine
0.75 μM
1.5 μM
3 μM
Tamoxifen
12.5 μM
25 μM
50 μM
MG132
75 nM
150 nM
300 nM
Bafilomycin
125 nM
250 nM
500 nM
Staurosporine
200 nM
350 nM
400 nM
n.s.
Q15_EV
Q15_mCherry
Q15_Mtf1

Supplementary Figure 4 - Secondary validation of mHTT suppressors

a Proliferation assay of the indicated cell lines. Bars indicate the mean ± SEM of 3 independent experiments, shown as dots. *P*-values were calculated with Two-way Repeated Measure ANOVA, comparing each candidate to the Q128_EV sample. **b** Expression analysis by qPCR of *Mtf1, Mt1, Mt2* genes confirmed upregulated expression in Q15_Mtf1 cells, compared to Q15_EV and Q15_mCherry cells. Bars indicate the mean ± SEM of 5 independent experiments shown as dots. Expression was normalised to the highest value. **c** Left panel: proliferation assay of WT mouse ESCs (E14 cells) transfected with an EV (E14_EV) or with a *Mtf1* encoding plasmid (E14_Mtf1). No significant changes on cell proliferation rate upon *Mtf1* overexpression was observed. Right panel: proliferation assay of Q15 cells transfected with an EV (Q15_EV) or with a *Mtf1* (Q15_Mtf1) encoding plasmid. No significant changes on cell proliferation rate upon *Mtf1* overexpression was observed. Bars indicate the mean ± SEM of 3 independent experiments shown as dots. *P*-values were calculated with Two-way Repeated Measure ANOVA. **d** CV quantification showing the number of surviving colonies in Q15_EV, Q15_mCherry and Q15_Mtf1 cell lines treated with stressors for 48 hours. Data were normalised to vehicle control (DMSO for Rotenone, 5-Azacytidine, MG132, Bafilomycin, Staurosporine; Ethanol for Cumene and Tamoxifen). Bars indicate the mean ± SEM of at least 3 independent experiments, shown as dots. n.s. indicates no statistically significant differences between Q15_Mtf1 and Q15_EV or Q15_mCherry, calculated with paired two-tailed *t*-test. Numerical values and all *P*-values are provided in the Source Data file.

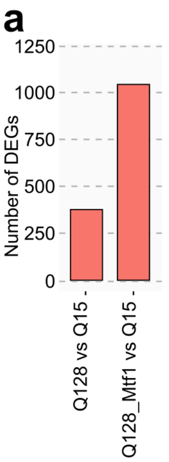

b

Enriched cathegory in Q128_Mtf1 vs Q128	*p*-value	Genes	Regulated in Q15_Mtf1 vs Q15	
transition metal ion transmembr. transporter (GO:0046915)	0.001	SLC25A37;SLC30A3;SLC30A2;SLC39A11; SLC39A10;SLC30A1;SLC39A4	YES	1.48e-02
zinc ion transport (GO:0006829)	0.003		YES	1.37e-02
Metal Ion SLC Transporters R-HSA-425410	0.009		YES	1.48e-02
cholesterol biosynthetic process (GO:0006695)	0.002	INSIG1;MSMO1;DHCR24;MVD; HMGCR;DHCR7;ACAT2	YES	1.64e-10
Cholesterol Biosynthesis R-HSA-191273	0.009		YES	2.43e-11
Metabolism Of Lipids R-HSA-556833	0.004	ARHGAP9;PECR;MTMR10;LPGAT1;MSMO1	YES	1.00e-03
fatty acid metabolic process (GO:0006631)	0.004	CPT1A;PLA2G1B;ACAD9;LPL;ACSL4;ACSL3	YES	1.76e-02
oxidoreductase activity, acting on the CH-CH (GO:0016628)	0.011	PECR;DHCR24;DHCR7;DECR2	YES	3.92e-02
triglyceride metabolic process (GO:0006641)	0.002	GK5;FABP3;LPGAT1;DGAT2;FABP5;APOC1	NO	2.12e-01
negative regulation of histone methylation (GO:0031061)	0.003	DNMT3B;EZHIP;MACROH2A1;AASS	NO	1.47e-01
glutathione transferase activity (GO:0004364)	0.011	GSTK1;GSTA3;ALOX5AP;GSTT2;PTGES	NO	7.89e-01
fatty acid synthase activity (GO:0004312)	0.012	ELOVL4;ELOVL2;OXSM	NO	7.79e-02
glutathione transmembr. transporter activity (GO:0034634)	0.023	ABCC1;GJA1	NO	8.79e-02
Glycogen Metabolism R-HSA-8982491	0.031	PHKG1;PHKG2;PYGM;PGM1	NO	2.37e-01
peroxisome organization (GO:0007031)	0.035	ABCD4;TMEM135;PEX6;PLAAT3	NO	2.61e-01
histone H3 deacetylation (GO:0070932)	0.037	HDAC3;SIRT6;HDAC8	NO	-

GO Biological Process   GO Molecular Function   Reactome2022

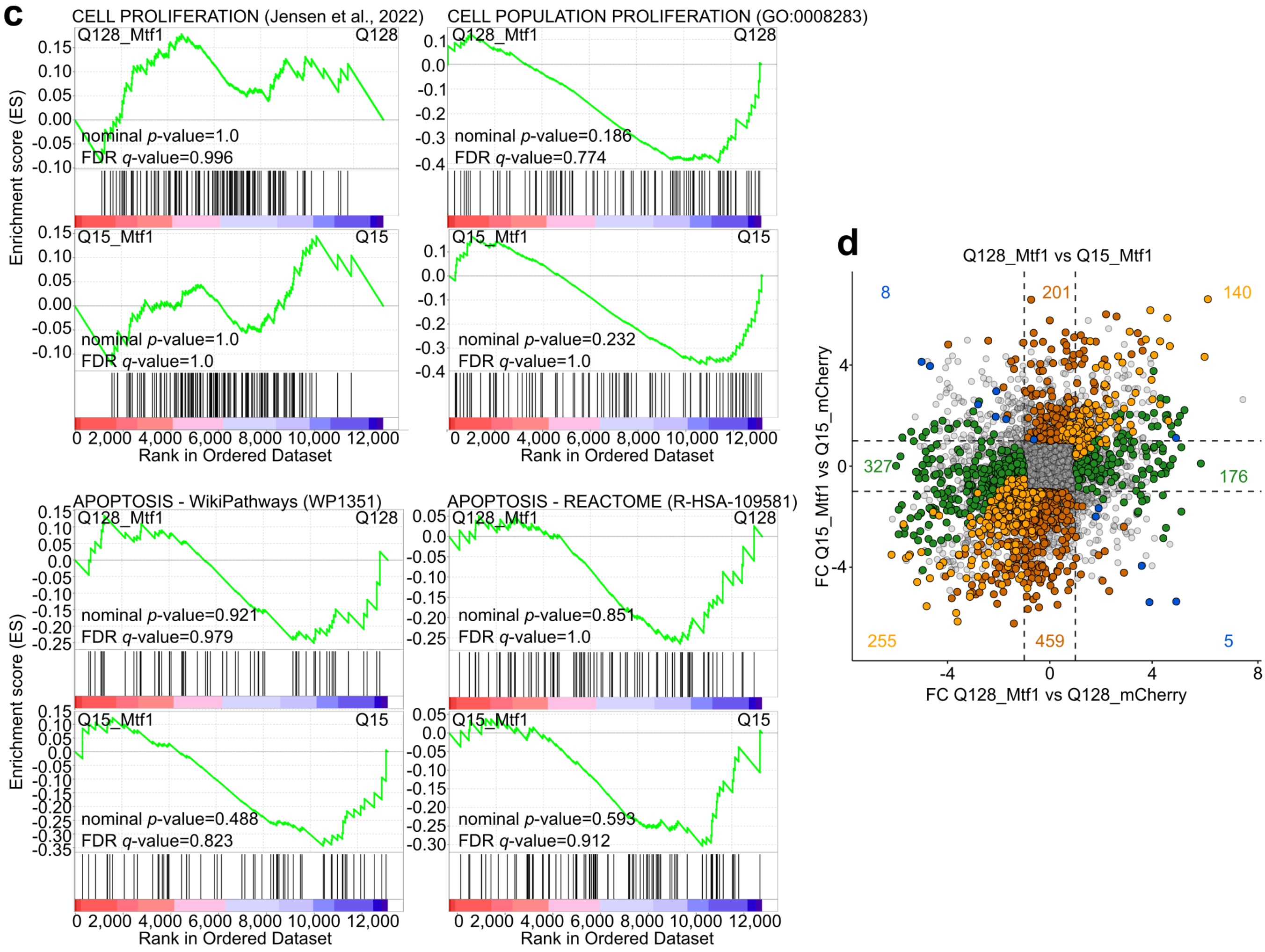

Supplementary Figure 5 - Processes regulated by Mtf1

a Bar plot showing the number of differentially expressed genes ($\log_2$ FC > 0.5 and *p*-value < 0.05, two-tailed Wald test) between Q128 cells and Q15 cells, or Q128_Mtf1 cells and Q15. 6 biological replicates for Q15, 7 biological replicates for Q128, and 6 biological replicates for Q128_Mtf1 cells were analysed. **b** Gene list enrichment analysis of genes significantly regulated by MTF1 in Q128 cells and Q15 cells. *P*-values were calculated by two-tailed Fisher Exact test using Enrichr database[154]. The first two columns indicate the processes enriched among genes regulated by MTF1 in Q128 cells, with the corresponding *p*-values. The third column shows genes belonging to the enriched processes. The fourth and fifth columns indicate whether the same processes were also enriched in genes controlled by MTF1 in Q15 cells, with the corresponding *p*-values. *P*-values above 0.05 were not considered statistically significant. **c** GSEA for genes regulated by MTF1 in Q128 cells or Q15 cells. Both nominal and adjusted *p*-values were calculated by GSEA software. **d** Analysis of genes differentially expressed in Q128 cells and Q15 cells upon expression of Mtf1. The *x* axis indicates the FC in expression between Q128_Mtf1 vs Q128_mCherry cells, the *y* axis indicates the FC in expression between Q15_Mtf1 vs Q15_mCherry cells. Green dots indicate genes differentially expressed (log2 FC > |1|, shown as dashed lines, *p*-value < 0.05, two tailed Wald test) only in Q128_Mtf1 vs Q128_mCherry. Brown dots indicate genes differentially expressed only in Q15_Mtf1 vs Q15_mCherry cells. Orange dots are genes regulated in both Q128 and Q15 cells by *Mtf1* in a coherent way in both cell lines. Blue dots show rare genes that are upregulated by *Mtf1* in Q128 cells and down regulated in Q15 cells, or vice versa. Grey dots indicate genes that are not differentially expressed. n=3 biological replicates for Q15, n=4 for Q15_Mtf1, n=4 for Q128 and n=3 for Q128_Mtf1 cells.

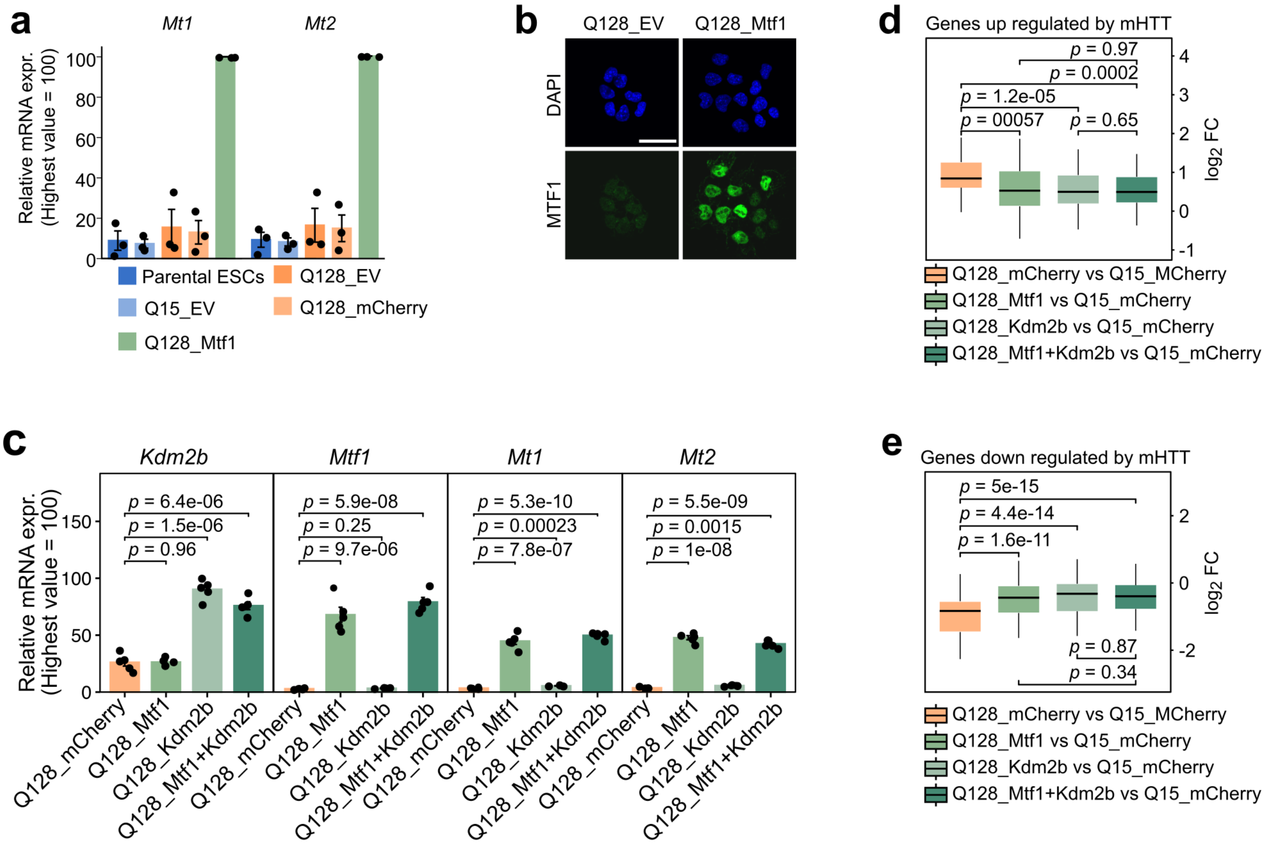
a
Mt1
Mt2
Relative mRNA expr.
(Highest value = 100)
100
80
60
40
20
0
Parental ESCs
Q128_EV
Q15_EV
Q128_mCherry
Q128_Mtf1
b
Q128_EV
Q128_Mtf1
DAPI
MTF1
d
Genes up regulated by mHTT
p = 0.97
p = 0.0002
p = 1.2e-05
p = 00057
p = 0.65
log2 FC
4
3
2
1
0
-1
Q128_mCherry vs Q15_MCherry
Q128_Mtf1 vs Q15_mCherry
Q128_Kdm2b vs Q15_mCherry
Q128_Mtf1+Kdm2b vs Q15_mCherry
c
Kdm2b
Mtf1
Mt1
Mt2
Relative mRNA expr.
(Highest value = 100)
150
100
50
0
p = 6.4e-06
p = 1.5e-06
p = 0.96
p = 5.9e-08
p = 0.25
p = 9.7e-06
p = 5.3e-10
p = 0.00023
p = 7.8e-07
p = 5.5e-09
p = 0.0015
p = 1e-08
Q128_mCherry
Q128_Mtf1
Q128_Kdm2b
Q128_Mtf1+Kdm2b
e
Genes down regulated by mHTT
p = 5e-15
p = 4.4e-14
p = 1.6e-11
p = 0.87
p = 0.34
log2 FC
2
0
-2
Q128_mCherry vs Q15_MCherry
Q128_Mtf1 vs Q15_mCherry
Q128_Kdm2b vs Q15_mCherry
Q128_Mtf1+Kdm2b vs Q15_mCherry

Supplementary Figure 6 - Mtf1 regulates HD-related processes

a Gene expression analysis by qPCR confirmed a strong upregulation of *Mt1* and *Mt2* genes in Q128_Mtf1 cells (green). Bars indicate the mean ± SEM of 3 independent experiments shown as dots. Expression was normalised to the highest value. **b** Immunofluorescence for MTF1 in Q128_Mtf1 and Q128_EV cells, representative of n=3 experiments. Nuclei were stained with DAPI. Scale bar, 30 µm. The endogenous MTF1 signal in Q128_EV cells is barely detectable. **c** Gene expression analysis by qPCR of *Mtf1*, *Kdm2b* confirmed increased levels of genes in corresponding cell lines in which they were overexpressed and a strong upregulation of *Mt1* and *Mt2* genes in Q15_Mtf1, Q128_Mtf1 and Q128_Mtf1+Kdm2b cells. Bars indicate the mean of 4 independent experiments shown as dots. Expression was normalised to the highest value. **d** Analysis of the genes upregulated in Q128 cells relative to Q15 cells (left or right box plots, respectively), identified in Fig. 1f. We calculated the FCs of the indicated 4 comparisons and found that expression of Mtf1 in Q128 cells significantly reduced the changes caused by mHTT (compare first and second boxplots). Kdm2b had a similar effect (compare first and third box plots), while the combined expression of Mtf1 and Kdm2b did not show any additive or synergistic effect, and was not significantly different from single Mtf1 or Kdm2b expression. *P*-value calculated with two-tailed *t*-test. Boxes indicate the 1st (Q1), 2nd and 3rd (Q3) quantile, the upper whisker indicates the InterQuartile Range (Q3-Q1). 3 biological replicates for Q15, 4 biological replicates for Q128, 3 biological replicates for Q128_Mtf1, 3 biological replicates for Q128_Kdm2b and 4 biological replicates for Q128_Mtf1+Kdm2b cells were analysed. **e** Same analyses as in panel d, performed on genes downregulated in Q128 cells relative to Q15 cells. Numerical values are provided in the Supplementary Data 3.

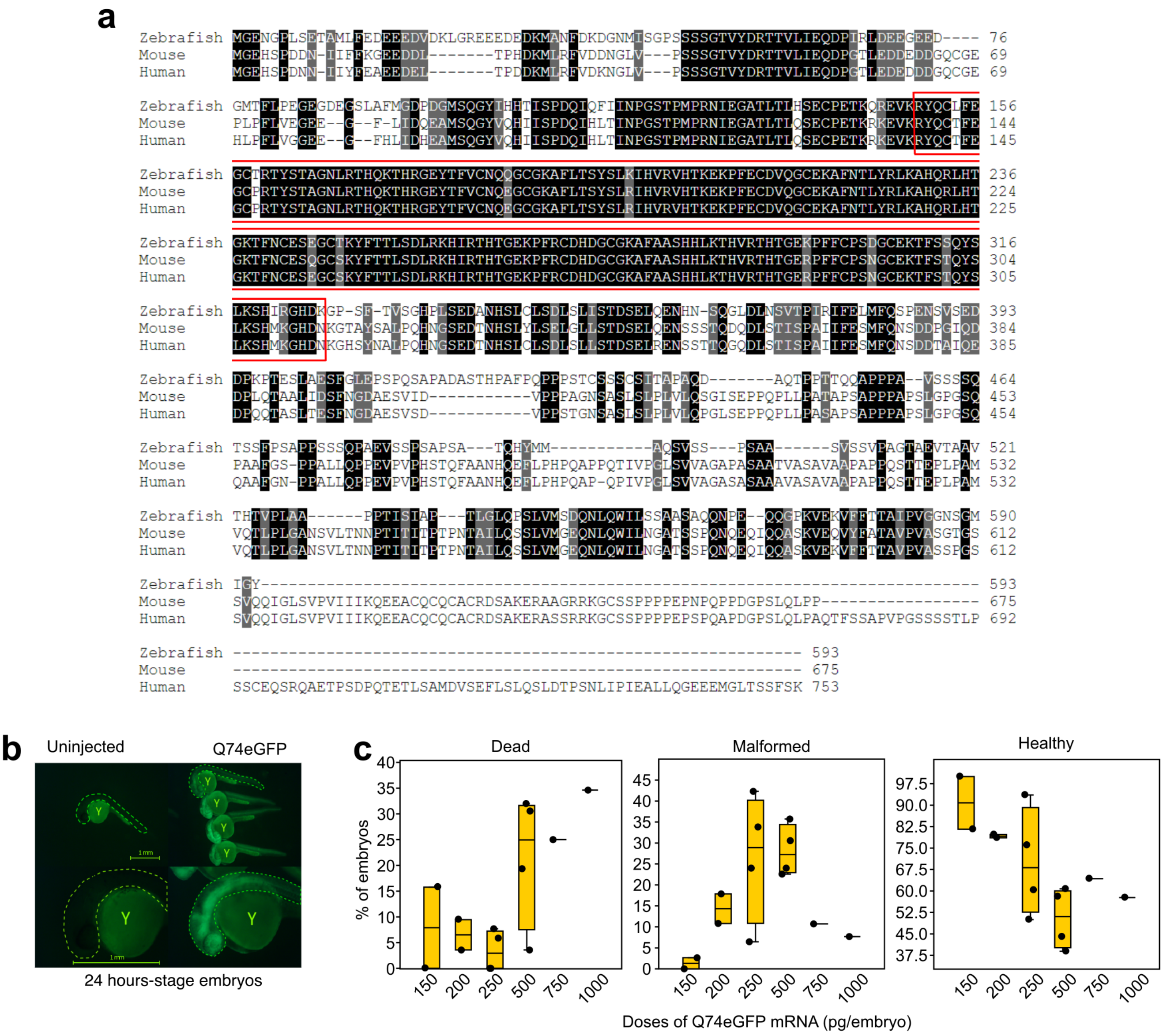
a
Zebrafish MGENGPLSETAMLFEDEEEDVDKLGREEEDEDKMANFDKDGNMISGPSSSSGTVYDRTTVLIEQDPIRLDEEGEED---- 76
Mouse MGEHSPDDN-IIFFKGEEDDL-------TPHDKMLRFVDDNGLV---PSSSGTVYDRTTVLIEQDPGTLEDDEDDGQCGE 69
Human MGEHSPDNN-IIYFEAEEDEL-------TPDDKMLRFVDKNGLV---PSSSGTVYDRTTVLIEQDPGTLEDEDDGQCGE 69
Zebrafish GMTFLPEGEGDEGSLAFMGDPDGMSQGYIHHTISPDQIQFIINPGSTPMPRNIEGATLTLHSECPETKQREVKRYQCLFE 156
Mouse PLPFLVEGEE--G--F-LIDQEAMSQGYVQHIISPDQIHLTINPGSTPMPRNIEGATLTLQSECPETKRKEVKRYQCTFE 144
Human HLPFLVGGEE--G--FHLIDHEAMSQGYVQHIISPDQIHLTINPGSTPMPRNIEGATLTLQSECPETKRKEVKRYQCTFE 145
Zebrafish GCTRTYSTAGNLRTHQKTHRGEYTFVCNQQGCGKAFLTSYSLKIHVRVHTKEKPFECDVQGCEKAFNTLYRLKAHQRLHT 236
Mouse GCPRTYSTAGNLRTHQKTHRGEYTFVCNQEGCGKAFLTSYSLRIHVRVHTKEKPFECDVQGCEKAFNTLYRLKAHQRLHT 224
Human GCPRTYSTAGNLRTHQKTHRGEYTFVCNQEGCGKAFLTSYSLRIHVRVHTKEKPFECDVQGCEKAFNTLYRLKAHQRLHT 225
Zebrafish GKTFNCESEGCTKYFTTLSDLRKHIRTHTGEKPFRCDHDGCGKAFAASHHLKTHVRTHTGEKPFFCPSDGCEKTFSSQYS 316
Mouse GKTFNCESQGCSKYFTTLSDLRKHIRTHTGEKPFRCDHDGCGKAFAASHHLKTHVRTHTGERPFFCPSNGCEKTFSTQYS 304
Human GKTFNCESEGCSKYFTTLSDLRKHIRTHTGEKPFRCDHDGCGKAFAASHHLKTHVRTHTGERPFFCPSNGCEKTFSTQYS 305
Zebrafish LKSHIRGHDKGP-SF-TVSGHPLSEDANHSLCLSDLSLISTDSELQENHN-SQGLDLNSVTPIRIFELMFQSPENSVSED 393
Mouse LKSHMKGHDNKGTAYSALPQHNGSEDTNHSLYLSELGLLSTDSELQENSSSTQDQDLSTISPAIIFESMFQNSDDPGIQD 384
Human LKSHMKGHDNKGHSYNALPQHNGSEDTNHSLCLSDLSLLSTDSELRENSSTTQGQDLSTISPAIIFESMFQNSDDTAIQE 385
Zebrafish DPKPTESLAESFGLEPSPQSAPADASTHPAFPQPPPSTCSSSCSITAPAQD-------AQTPPTTQQAPPPA--VSSSSQ 464
Mouse DPLQTAALIDSFNGDAESVID-----------VPPPAGNSASLSLPLVLQSGISEPPQPLLPATAPSAPPPAPSLGPGSQ 453
Human DPQQTASLTESFNGDAESVSD-----------VPPSTGNSASLSLPLVLQPGLSEPPQPLLPASAPSAPPPAPSLGPGSQ 454
Zebrafish TSSFPSAPPSSSQPAEVSSPSAPSA---TQHYMM-----------AQSVSS---PSAA------SVSSVPAGTAEVTAAV 521
Mouse PAAFGS-PPALLQPPEVPVPHSTQFAANHQEFLPHPQAPPQTIVPGLSVVAGAPASAATVASAVAAPAPPQSTTEPLPAM 532
Human QAAFGN-PPALLQPPEVPVPHSTQFAANHQEFLPHPQAP-QPIVPGLSVVAGASASAAAVASAVAAPAPPQSTTEPLPAM 532
Zebrafish THTVPLAA------PPTISIAP---TLGLQPSLVMSDQNLQWILSSAASAQQNPE--QQGPKVEKVFFTTAIPVGGNSGM 590
Mouse VQTLPLGANSVLTNNPTITITPTPNTAILQSSLVMGEQNLQWILNGATSSPQNQEQIQQASKVEQVYFATAVPVASGTGS 612
Human VQTLPLGANSVLTNNPTITITPTPNTAILQSSLVMGEQNLQWILNGATSSPQNQEQIQQASKVEKVFFTTAVPVASSPGS 612
Zebrafish IGY--- 593
Mouse SVQQIGLSVPVIIIKQEEACQCQCACRDSAKERAAGRRKGCSSPPPPEPNPQPPDGPSLQLPP----------------- 675
Human SVQQIGLSVPVIIIKQEEACQCQCACRDSAKERASSRRKGCSSPPPPEPSPQAPDGPSLQLPAQTFSSAPVPGSSSSTLP 692
Zebrafish -- 593
Mouse -- 675
Human SSCEQSRQAETPSDPQTETLSAMDVSEFLSLQSLDTPSNLIPIEALLQGEEEMGLTSSFSK 753
b
Uninjected
Q74eGFP
Y
1mm
24 hours-stage embryos
c
Dead
Malformed
Healthy
% of embryos
0
5
10
15
20
25
30
35
40
45
37.5
45.0
52.5
60.0
67.5
75.0
82.5
90.0
97.5
150
200
250
500
750
1000
Doses of Q74eGFP mRNA (pg/embryo)

Supplementary Figure 7 - Mtf1 counteracts mHTT effects in zebrafish

a Amino acid sequence alignment of zebrafish, mouse and human MTF1. Shaded black and grey colour shows sequence identity and similarity. The zinc finger domain is highlighted in the red box. **b** Representative images obtained by fluorescent microscopy of 24 hours-stage embryos injected with Q74eGFP mRNA (250 pg/embryo, right panels) compared to uninjected controls (left panels). Dashed lines represent the region of interest, while the yolk (Y) shows autofluorescence. Q74eGFP mRNA-injected embryos exhibited a fluorescent signal along the entire embryo. Same results were obtained in n=8 independent injection experiments. **c** Percentages of dead, malformed and healthy embryos phenotypically scored 24 hpf and obtained after injecting increasing doses of Q74eGFP mRNA, ranging from 150 to 1000 pg/embryo. Doses above 500 pg/embryo were highly toxic for embryos, leading many fish to die, while doses below 500 pg/embryo were more tolerated. The dose of 250 pg/embryo showed the highest rate of malformations with the lowest level of death, and for this reason it was chosen for the following experiments. Each dot represents an independent experiment. Box plots indicate 1st, 2nd and 3rd quantile, whiskers indicate minimum and maximum values.

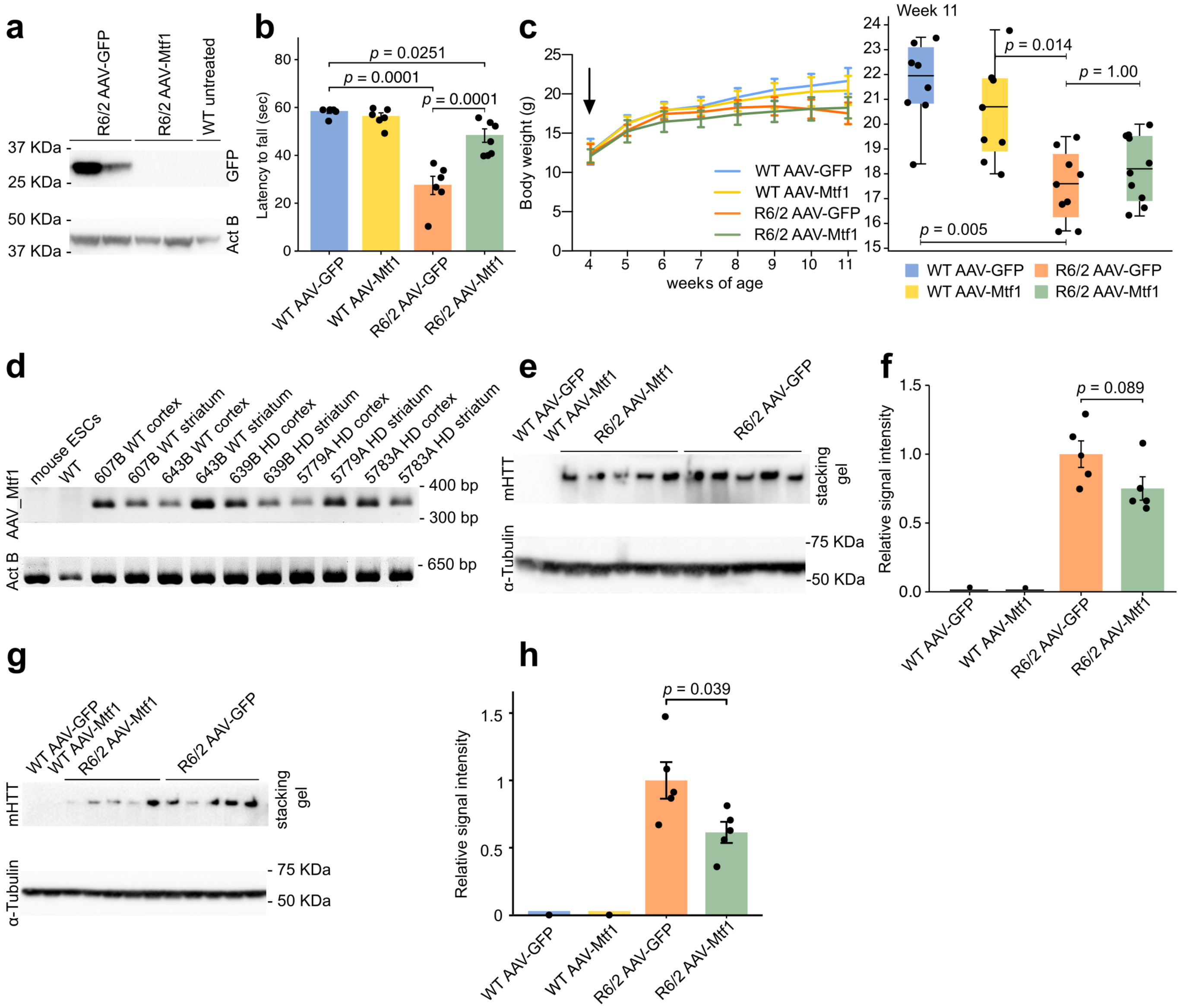

a
R6/2 AAV-GFP
R6/2 AAV-Mtf1
WT untreated
37 KDa
25 KDa
50 KDa
37 KDa
GFP
Act B
b
Latency to fall (sec)
p = 0.0251
p = 0.0001
p = 0.0001
WT AAV-GFP
WT AAV-Mtf1
R6/2 AAV-GFP
R6/2 AAV-Mtf1
c
Body weight (g)
weeks of age
WT AAV-GFP
WT AAV-Mtf1
R6/2 AAV-GFP
R6/2 AAV-Mtf1
Week 11
p = 0.014
p = 1.00
p = 0.005
d
mouse ESCs
WT
607B WT cortex
607B WT striatum
643B WT cortex
643B WT striatum
639B HD cortex
639B HD striatum
5779A HD cortex
5779A HD striatum
5783A HD cortex
5783A HD striatum
AAV_Mtf1
Act B
400 bp
300 bp
650 bp
e
WT AAV-GFP
WT AAV-Mtf1
R6/2 AAV-Mtf1
R6/2 AAV-GFP
mHTT
stacking gel
α-Tubulin
75 KDa
50 KDa
f
Relative signal intensity
p = 0.089
g
WT AAV-GFP
WT AAV-Mtf1
R6/2 AAV-Mtf1
R6/2 AAV-GFP
mHTT
stacking gel
α-Tubulin
75 KDa
50 KDa
h
Relative signal intensity
p = 0.039

Supplementary Figure 8 - AAV-vector delivery of Mtf1 alleviates motor deficit in R6/2 mice

a Western Blot on brain lysate of 11 week old R6/2 mice tail-vein injected with AAV-containing GFP. AAV-Mtf1 injected mice were used as negative control. Act B (*β*-actin) was used as loading control. N=1 experiment. **b** Motor performance assessed by the Rotarod test of the indicated experimental groups at 8 weeks of age. Number of injected mice: WT AAV-GFP = 5; WT AAV-Mtf1 = 5; R6/2 AAV-GFP = 5; R6/2 AAV-Mtf1 = 5. *P*-values were calculated with the unpaired two-tailed Mann-Whitney U test with Bonferroni correction. **c** Average body weight of R6/2 and WT mice after viral injection with either AAV-GFP or AAV-Mtf1. Left: line plots show mean ± SD of each experimental group at each time point. WT AAV-GFP vs R6/2 AAV-GFP *p*-value < 0.0001; R6/2 AAV-Mtf1 vs R6/2 AAV-GFP *p*-value = 0.0350, *p*-values were calculated with the Two-way repeated measure ANOVA. Right: boxplots plots indicate 1st, 2nd and 3rd quartile; whiskers indicate minimum and maximum. Number of injected mice: WT AAV-GFP = 8; WT AAV-Mtf1 = 9; R6/2 AAV-GFP = 9; R6/2 AAV-Mtf1 = 10. *P*-values were calculated with the unpaired two-tailed Mann-Whitney U test with Bonferroni correction. **d** PCR on total DNA. Act B (*β*-actin) was used as a positive control for PCR reaction. n=3 independent experiments. **e** Cropped western blotting for EM48-positive SDS-insoluble mHTT aggregates in cortex lysate from the indicated samples at 11 weeks of age. n=1 experiment. **f** Quantification of signal intensity of EM48-positive SDS-insoluble mHTT aggregates. Bars indicate the mean ± SD. **g** Western blotting for mHTT aggregates in striatal lysate at 11 weeks of age. N=1 experiment. **h** Quantification of mHTT aggregates. Bars indicate the mean ± SD. Number of mice used in **e-h**: WT AAV-GFP = 1; WT AAV-Mtf1 = 1; R6/2 AAV-GFP = 5; R6/2 AAV-Mtf1 = 5. Unpaired two-tailed t-test.

Uncropped gels and numerical values are provided in the Source data file.

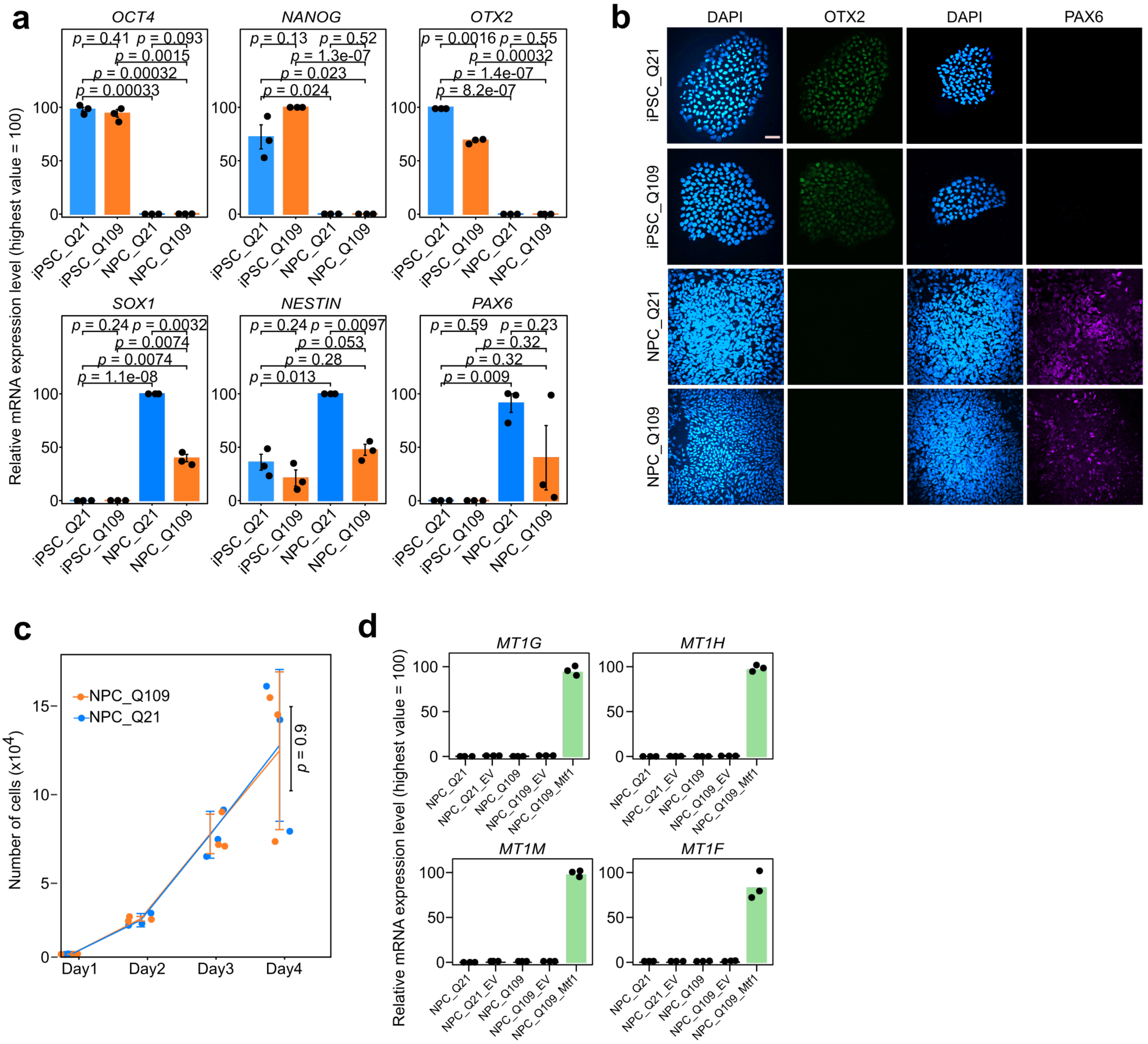

a
OCT4
NANOG
OTX2
SOX1
NESTIN
PAX6
Relative mRNA expression level (highest value = 100)
iPSC_Q21
iPSC_Q109
NPC_Q21
NPC_Q109
b
DAPI
OTX2
DAPI
PAX6
c
NPC_Q109
NPC_Q21
Number of cells (x10^4)
Day1
Day2
Day3
Day4
p = 0.9
d
MT1G
MT1H
MT1M
MT1F
NPC_Q21_EV
NPC_Q109_EV
NPC_Q109_Mtf1

Supplementary Figure 9 - MTF1 rescues mHTT-dependent alterations in human NPCs

a Gene expression analysis by qPCR of pluripotency genes POU5F1 (also known as *OCT4)*, *NANOG, OTX2* and early neural markers *SOX1*, *NES* (also known as *NESTIN*), *PAX6* in iPSC_Q21 and iPSC_Q109 cells, compared to NPCs. Results show elevated expression levels of pluripotency markers in iPCSs, compared to NPCs. NPCs do not retain pluripotency and express early neural markers. Bars indicate the mean ± SEM of 3 independent experiments shown as dots. Expression was normalised to the highest value. **b** Immunofluorescence for pluripotency marker OTX2 and early neural marker PAX6 in iPSC_Q21, iPSC_Q109, NPC_Q21, NPC_Q109. Scale bar, 40 µm. Similar results were obtained in n=3 independent experiments. **c** Proliferation assay of NPC_Q109 (orange) and NPC_Q21 (blue) cells showed comparable cell proliferation. Bars indicate the mean ± SEM of 3 independent experiments, shown as dots. *P*-value was calculated with Two-way Repeated Measure ANOVA. **d** Gene expression analysis by qPCR of metallothioneins (*MT1G*, *MT1H*, *MT1M*, *MT1F*) in NPCs Q21_Mtf1 and Q109_Mtf1 compared to NPCs Q21, Q109 and Q21_EV, Q109_EV. Results show strong induction of metallothioneins in NPCs Q21 and Q109 transfected with *Mtf1*. Expression was normalised to the highest value. N = 3 technical replicates, shown as dots. Numerical values are provided in the Source data file.

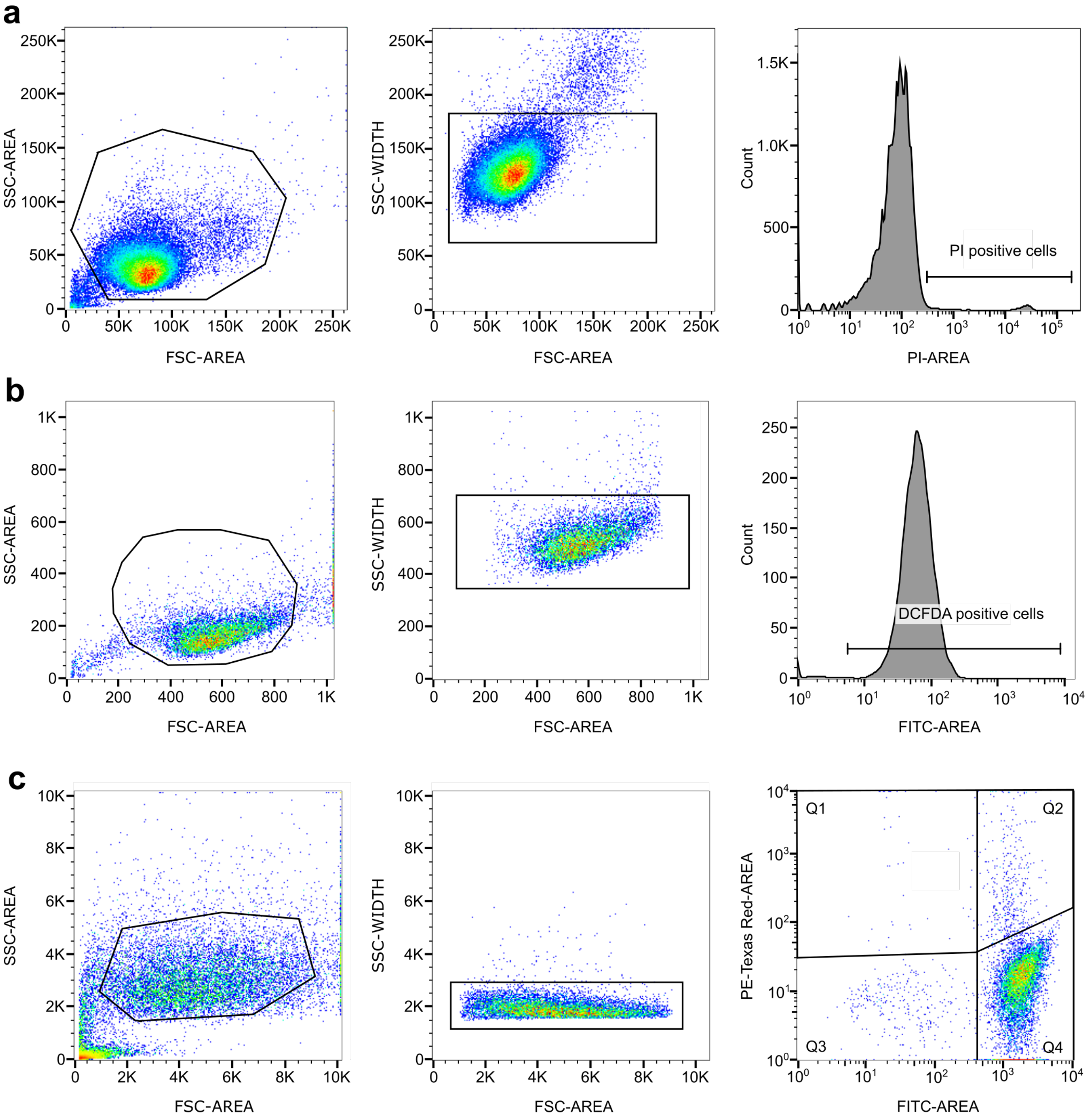
a
SSC-AREA
FSC-AREA
SSC-WIDTH
Count
PI positive cells
PI-AREA
b
DCFDA positive cells
FITC-AREA
c
Q1
Q2
Q3
Q4
PE-Texas Red-AREA

Supplementary Figure 10 – Gating strategy

a Representative gating strategy to evaluate cell death, measured as Propidium iodide (PI) uptake, in mouse ESCs by flow cytometry. First, cell population was distinguished from cellular debris (left panel). Singlets were chosen (centre panel), followed by gating of PI positive cells (right panel). This gating strategy corresponds to Fig. 1d and Fig. 5e. **b** Representative gating strategy to evaluate ROS content, measured as 2′,7′-dichlorodihydrofluorescein diacetate (H_2DCFDA) fluorescence, in mouse ESCs by flow cytometry. First, cell population was distinguished from cellular debris (left panel). Singlets were chosen (centre panel), followed by gating of DCFDA positive cells (right panel). This gating strategy corresponds to Fig. 1e and Fig. 5f. **c** ERepresentative gating strategy to evaluate either ROS content, measured as 2′,7′-dichlorodihydrofluorescein diacetate (H_2DCFDA) fluorescence (Fig. 8e), or cell death by Annexin V uptake (Fig. 8f), in human NPCs transfected with an mCherry vector by flow cytometry. First, cell population was distinguished from cellular debris (left panel). Singlets were chosen (centre panel), followed by gating of double positive cells in Q2 (right panel).

Supplementary Table 1. – PCR primers

**Splinkerette-adaptor primers**	**Sequence**
AdapterA hairpin strand with 5'-GATC overhang	GATCCCACTAGTGTCGACACCAGTCTCTAATTTTTTTTTTCAAAAAAA
AdapterB linear strand	CGAAGAGTAACCGTTGCTAGGAGAGACCGTGGCTGAATGAGACTGGT GTCGACACTAGTGG
Adapter PCR1	CGAAGAGTAACCGTTGCTAG GAGAGACC
Adapter PCR2	GTGGCTGAATGAGACTGGTGTCGAC
**piggyBac primers**	**Sequence**
pB3'-ITR PCR1	CAGTGACACTTACCGCATTGACAAGCACGC
pB5'-ITR PCR1	CCTCGATATACAGACCGATAAAACACATGC
pB3'-ITR PCR2	GAGAGAGCAATATTTCAAGAATGCATGCGT
pB5'-ITR PCR2	ACGCATGATTATCTTTAACGTACGTCACAA
ITR, piggyBac inverted terminal repeats	
**h-iPSCs genotyping primers**	**Sequence**
CAG1	ATG AAG GCC TTC GAG TCC CTC AAG TCC TTC
HU3	GGC GGC TGA GGA AGC TGA GGA

ITR, piggyBac inverted terminal repeats

Supplementary Table 2. – Antibodies

Antibody	Species	Source	Dilution	Validation
HTT	Mouse monoclonal (clone 1HU-4C8)	Millipore cat. MAB2166	WB 1:5000	Macdonald, D. et al., PloS one 9(5): e96854 (2014)
HTT	Mouse monoclonal (clone EM48)	Millipore cat. MAB5374	WB 1:1000 IF 1:50	Wang, C.E., et al., J. Cell Biol., 181, 803–816 (2008)
GAPDH	Mouse monoclonal (clone 6C5)	Millipore cat. MAB374	WB 1:5000	Yao, X. et al., PloS one 10, e0139416 (2015)
GFP	Species independent	Abcam cat. Ab290	WB 1:1000	Zhou, Y.Q. et al., EMBO Mol Med 12, e10233 (2020)
α-TUBULIN	Mouse monoclonal (clone B-5-1-2)	Sigma-Aldrich T5168	WB 1:5000	Kalebic, N., et al., Mol Cell Bio 1114–1123 (2013)
β-ACTIN	Mouse monoclonal (clone 8H10D10)	Cell Signaling Technology cat. 3700	WB 1:2000	Condeelis, J. et al., Trends Cell Biol 11, 288-93 (2001)
MTF1	Rabbit polyclonal	Novus biologicals cat. NBP1-86380	IF 1:200	Zhang, D. et al., J. Agric. Food Chem 67, 4611-4622 (2019)
OCT4	Mouse monoclonal (clone C-10)	Santa Cruz SC-5279	IF 1:300	Szlachcic, W. J. et al., Front Mol Neurosci 8;10:253 (2017)
NANOG	Rabbit monoclonal (clone D73G4)	Cell Signalling Technology D73G4	IF 1:100	Zorzan I. et al., Nat Comm 11,2364 (2020)
PAX6	Rabbit polyclonal (clone Poly19013)	Biolegend 901302	IF 1:300	Ooi J. et al., Cell Rep. 26:2494 (2019)
SOX1	Goat polyclonal	R&D AF3369-SP	IF 1:50	Malankhanova T. et al., J Pers Med 10(4):215 (2020)
NESTIN	Mouse monoclonal (clone 10C2)	Sigma-Aldrich MAB5326	IF 1:500	Ooi J, et al., Cell Rep. 26:2494 (2019)
OTX2	Goat polyclonal	R&D AF1979	IF 1:100	Malankhanova T. et al., J Pers Med 10(4):215 (2020)
Alexa Fluor 568	Donkey anti- Mouse IgG (H+L)	Invitrogen A-10037	IF 1:500	Zorzan I. et al., Nat Comm 11,2364 (2020)
Alexa Fluor 647	Donkey anti- Rabbit IgG (H+L)	Invitrogen A-31573	IF 1:500	Zorzan I. et al., Nat Comm 11,2364 (2020)
Alexa Fluor 488	Donkey anti- Goat IgG (H+L)	Invitrogen A-11055	IF 1:500	Zorzan I. et al., Nat Comm 11,2364 (2020)

Supplementary Table 3. – qPCR primers

Organism	Gene	Forward primer sequence	Reverse primer sequence
Mouse	Arid1b	CTGGACCTGTTCCGACTGTA	CAACGTTCAGGTTGGTTGC
Mouse	Fbxo34	CCTCCCAGTCTTGAGTCAGC	CATAGGCGTTCTCAGGGTGT
Mouse	Gapdh	ATCCTGCACCACCAACTGCT	GGGCCATCCACAGTCTTCTG
Human/Mouse	Htt	TGATGGATTCTAATCTTCCAAGG	GTGAGCCAGCTCAGCAAAC
Mouse	Kdm2b	CTGCTCTCGAAGCTCCAACT	TTTCCACAGCGTTTGAAGAA
Mouse	Kdm5b	ACCTGACCTCCGACACAAAG	TGGCTTCTGTTGCCTCTTCT
Mouse	Kdm5b - UTR	ATCTGTGTGCGCTGTACTGG	GCGATGCAATTTCCTCATTT
Mouse	Mt1	CTGCTGCTCCTGCTGTCC	CAGCGCTGTTCGTCACAT
Mouse	Mt2	GCAAATGCAAACAATGCAAA	CACTTGTCGGAAGCCTCTTT
Mouse	Mtf1	GAAGCAATGTCCCAGGGTTA	CTCCTCGGTGAGTCTTCTGG
	AAV_Mtf1	AGCAGATTGGCCTCAGTGTT	TCCATCACTAGGGGTTCCTG
Mouse	β-Actin	GTTGACATCCGTAAAGACC	TGGAAGGTGGACAGTGAG
Mouse	Synj2	CAGGAGGCAGAAGCAGCTAT	TGGCTTAGAAGACCCTTGGTT
	eGFP	TATATCATGGCCGACAAGCA	ACTGGGTGCTCAGGTAGTGG
Zebrafish	B2m	GCCTTCACCCCAGAGAAAGG	CCGGTTTGGATTTACATGTTG
Zebrafish	Eef1a	GACAAGAGAACCATCGAG	CTGAAACTCACCGACAC
Zebrafish	Gapdh	GTGGAGTCTACTGGTGTCTTC	GTGCAGGAGGCATTGCTTACA
Zebrafish	Tuba1b	CCCACTGTCATTGATGAGG	CGAACCCTATCTAAAACAAGGTCA
Human	OCT4	GTGGAGGAAGCTGACAACAA	ATTCTCCAGGTTGCCTCTCA
Human	NANOG	CAAAGGCAAACAACCCACTT	TCTGCTGGAGGCTGAGGTAT
Human	PAX6	TGGGCAGGTATTACGAGACTG	ACTCCCGCTTATACTGGGCTA
Human	SOX1	GCTGACACCAGACTTGGGTTT	CCCCTCGAGCAAAGAAAACG
Human	NESTIN	TGGCAAAGGAGCCTACTCCAAGAA	ATCGGGATTCAGCTGACTTAGCCT
Human	OTX2	CAAAGTGAGACCTGCCAAAAAGA	TGGACAAGGGATCTGACAGTG
Human	GAPDH	CGAGATCCCTCCAAAATCAA	GGCAGAGATGATGACCCTTT
Human	MT1A	ATGGACCCCAACTGCTCCT	CAGCTGCACTTCTCTGATGC
Human	MT1B	ATGGATCCCAACTGCTCCT	AAGAGCAGCAGCACTTCTTG
Human	MT2A	CGCCTCTTCAGCACGCCATGGATCCCAAC	CATCAGGCGCAGCAGCTGCAC
Human	MT1G	AGGTGTCTCCTGCACCTG	ACTTGGCACAGCCCACAG
Human	MT1H	TCCTGCAAGTGCAAAAAGTG	TGACGCCCCTTTGCAGAT
Human	MT1M	CAACTGCTCCTGCACCACT	AGCTGCAGTTCTCCAACGTC
Human	MT1F	AGTCTCTCCTCGGCTTGC	ACATCTGGGAGAAAGGTTGTC

Supplementary Table 4. – Mice list

Age	Treatment	ID code	Gender	Body weight	Brain weigth	Latency to fall (Rotarod)	HLT	Clasping	HTT aggregates (WB e IHC)	DHE (IHC)
11 weeks	WT_GFP	630B	♀	x		x	x			
	WT_GFP	616B	♀	x		x	x			
	WT_GFP	640B	♀	x		x	x			
	WT_GFP	5777A	♀	x		x	x			
	WT_GFP	5765A	♀	x		x	x			
	WT_GFP	5770A	♀	x		x	x			
	WT_GFP	5789A	♀	x		x	x			
	WT_GFP	596B	♀	x		x	x			
	WT_MTF1	644B	♀	x		x	x			
	WT_MTF1	598B	♀	x		x	x			
	WT_MTF1	5805A	♀	x		x	x			
	WT_MTF1	5806A	♀	x		x	x			
	WT_MTF1	5780A	♀	x		x	x			
	WT_MTF1	5804A	♀	x		x	x			
	WT_MTF1	643B	♀	x		x	x			
	WT_MTF1	607B	♀	x		x	x			
	WT_MTF1	618B	♀	x		x	x			
	R6/2_GFP	605B	♀	x		x	x		x	
	R6/2_GFP	610B	♀	x		x	x			
	R6/2_GFP	615B	♀	x		x	x			
	R6/2_GFP	621B	♀	x		x			x	
	R6/2_GFP	597B	♀	x		x	x		x	
	R6/2_GFP	5766A	♀	x		x	x		x	
	R6/2_GFP	5781A	♀	x		x	x		x	
	R6/2_GFP	5769A	♀	x		x	x			
	R6/2_GFP	5778A	♀	x		x	x			
	R6/2_MTF1	637B	♀	x		x	x		x	
	R6/2_MTF1	638B	♀	x		x	x		x	
	R6/2_MTF1	639B	♀	x		x	x			
	R6/2_MTF1	617B	♀	x		x	x		x	
	R6/2_MTF1	612B	♀	x		x	x			
	R6/2_MTF1	5782A	♀	x		x	x		x	
	R6/2_MTF1	5783A	♀	x		x	x			
	R6/2_MTF1	5784A	♀	x		x	x		x	
	R6/2_MTF1	5779A	♀	x		x	x			
	R6/2_MTF1	5803A	♀	x		x	x			
8 weeks	WT_GFP	ID 2014E	♀	x	x	x		x		x
	WT_GFP	ID 1999E	♀	x	x	x		x		x
	WT_GFP	ID 2018E	♀	x	x	x		x		x
	WT_MTF1	ID 2002E	♀	x	x	x		x		x
	WT_MTF1	ID 2003E	♀	x	x	x		x		x
	WT_MTF1	ID 2012E	♀	x	x	x		x		x
	R6/2_GFP	ID 2010E	♀	x	x	x		x	x	x
	R6/2_GFP	ID 2000E	♀	x	x	x		x	x	x
	R6/2_GFP	ID 2019E	♀	x	x	x		x	x	x
	R6/2_MTF1	ID 2004E	♀	x	x	x		x	x	x
	R6/2_MTF1	ID 2007E	♀	x	x	x		x	x	x
	R6/2_MTF1	ID 2008E	♀	x	x	x		x	x	x
7 weeks	WT_GFP	ID 2107E	♀	x	x	x		x		x
	WT_GFP	ID 2108E	♀	x	x	x		x		
	WT_GFP	ID 2117E	♀	x	x	x		x		
	WT_MTF1	ID 2111E	♀	x	x	x		x		x
	WT_MTF1	ID 2112E	♀	x	x	x		x		
	WT_MTF1	ID 2115E	♀	x	x	x		x		
	R6/2_GFP	ID 2103E	♀	x	x	x		x	x	x
	R6/2_GFP	ID 2104E	♀	x	x	x		x		
	R6/2_GFP	ID 2124E	♀	x	x	x		x		
	R6/2_MTF1	ID 2113E	♀	x	x	x		x	x	x
	R6/2_MTF1	ID 2154E	♀	x	x	x		x		
	R6/2_MTF1	ID 2171E	♀	x	x	x		x		
	R6/2_MTF1	ID 2160E	♂	x	x	x		x		
