# Supplementary material for: Genome-wide screening in pluripotent cells identifies Mtf1 as a suppressor of mutant huntingtin toxicity
Source: Nat Commun. 2023 Jul 5;14:3962. doi: 10.1038/s41467-023-39552-9 (PMC10322923; doi:10.1038/s41467-023-39552-9)
Supplement: Supplementary file 3 — Description of Additional Supplementary Files [file 41467_2023_39552_MOESM3_ESM.pdf]

Description of Additional Supplementary Files

File Name: Supplementary Data 1
Description: This table reports RNAseq data of following samples: Q15_mCherry; Q128_mCherry; Q128_Mtf1; Q128_Kdm2b; Q128_Mtf1+Kdm2b; Q15_Mtf1. Table reports Count per Millions in each sample and differential analysis for the following comparisons: Q128 vs Q15; Q128_Mtf1 vs Q128; Q128_Mtf1 vs Q15. For differential expression analysis not adjusted p-values were calculated with two-tailed Wald test . At least 3 biological replicates analysed for each condition.

File Name: Supplementary Data 2
Description: This table reports differential expression analysis for the following comparisons: Q128_Mtf1 vs Q128_mCherry; Q15_Mtf1 vs Q15_mCherry. For differential analysis not adjusted p-values were calculated with two-tailed Wald test. Number of biological replicates included in the analysis: n = 4 for Q128, n = 3 for Q15, n = 3 for Q128_Mtf1, n = 4 for Q15_Mtf1. Differentially expressed genes are defined according to the following thresholds: log2 FC > |1|, p-value < 0.05. Genes in green are differentially expressed only in Q128_Mtf1 vs Q128_mCherry. Genes in brown are differentially expressed only in Q15_Mtf1 vs Q15_mCherry cells. Genes in orange are regulated in both Q128_mCherry and Q15_mCherry cells by Mtf1 in a coherent way (e.g. upregulated in both cell lines). Genes in blue are upregulated by Mtf1 in Q128 cells and down regulated in Q15, or viceversa.

File Name: Supplementary Data 3
Description: This table reports differential expression analysis for the following comparisons: Q128_mCherry vs Q15_mCherry; Q128_Mtf1 vs Q15_mCherry; Q128_Kdm2b vs Q15_mCherry; Q128_Mtf1+Kdm2b vs Q15_mCherry. For differential expression analysis not adjusted p-values were calculated with two-tailed Wald test. Number of biological replicates included in the analysis: n = 4 for Q128, n = 3 for Q15, n = 3 for Q128_Mtf1, n = 3 for Q128_Kdm2b and n = 4 for Q128_Mtf1+Kdm2b.
