# Supplementary material for: Genome-wide screening in pluripotent cells identifies Mtf1 as a suppressor of mutant huntingtin toxicity
Source: Nat Commun. 2023 Jul 5;14:3962. doi: 10.1038/s41467-023-39552-9 (PMC10322923; doi:10.1038/s41467-023-39552-9)
Supplement: Supplementary file 7 — Reporting Summary [file 41467_2023_39552_MOESM7_ESM.pdf]

Corresponding author(s): Graziano Martello

Last updated by author(s): Jun 6, 2023

# Reporting Summary

Nature Portfolio wishes to improve the reproducibility of the work that we publish. This form provides structure for consistency and transparency in reporting. For further information on Nature Portfolio policies, see our Editorial Policies and the Editorial Policy Checklist.

## Statistics

For all statistical analyses, confirm that the following items are present in the figure legend, table legend, main text, or Methods section.

| n/a | Confirmed | |
|---|---|---|
| ☐ | ☒ | The exact sample size (*n*) for each experimental group/condition, given as a discrete number and unit of measurement |
| ☐ | ☒ | A statement on whether measurements were taken from distinct samples or whether the same sample was measured repeatedly |
| ☐ | ☒ | The statistical test(s) used AND whether they are one- or two-sided<br>*Only common tests should be described solely by name; describe more complex techniques in the Methods section.* |
| ☒ | ☐ | A description of all covariates tested |
| ☐ | ☒ | A description of any assumptions or corrections, such as tests of normality and adjustment for multiple comparisons |
| ☐ | ☒ | A full description of the statistical parameters including central tendency (e.g. means) or other basic estimates (e.g. regression coefficient) AND variation (e.g. standard deviation) or associated estimates of uncertainty (e.g. confidence intervals) |
| ☐ | ☒ | For null hypothesis testing, the test statistic (e.g. *F*, *t*, *r*) with confidence intervals, effect sizes, degrees of freedom and *P* value noted<br>*Give P values as exact values whenever suitable.* |
| ☒ | ☐ | For Bayesian analysis, information on the choice of priors and Markov chain Monte Carlo settings |
| ☒ | ☐ | For hierarchical and complex designs, identification of the appropriate level for tests and full reporting of outcomes |
| ☒ | ☐ | Estimates of effect sizes (e.g. Cohen's *d*, Pearson's *r*), indicating how they were calculated |

*Our web collection on statistics for biologists contains articles on many of the points above.*

## Software and code

Policy information about availability of computer code

| | |
|---|---|
| Data collection | For qPCR data collection the Applied BiosystemsTM QuantStudio'" 6&7 Flex Real-Time PCR System version 1.0 and 1.3 was used.<br>For RNA quantification Qubit 4.0 was used.<br>mES western blots and iPSCs genotyping were digitally acquired by ImageQuant LAS 4000 or VWR Imager CHEMI Premium.<br>Mouse western blots were digitally acquired by ChemiDoc XRS+ (model n°: Universal Hood II) with Image Lab Software, BioRad (v. 6.1).<br>iPSCs and NPCs morphology data were collected with microscope Zeiss Axio Vert A1 FL-LED.<br>BD FACSCantoTM II and BIO-RAD S3e Cell Sorter cytometer were used for flow cytometry experiments.<br>For mESCs, iPSCs and NPCs immunostaining data Leica TCS SP5 LAS AF and Zeiss LSM900 Airyscan2 confocal microscopes were used.<br>For RNAseq, NovaSeq 6000 Sequencing System was used.<br>Metal data collection was performed with Inductively Coupled Plasma – Optical Emission Spectrometry (ICP-OES, mod 5110, Agilent).<br>For zebrafish larvae whole mount stainings data collection the Leica M165FC fluorescence microscope was used.<br>For mouse immunohistochemistry data collection, Nikon ECLIPSE Ni microscope was used. |
| Data analysis | Image analysis was performed with FIJI (v. 2.0.0 and v. 2.9.0) and NIS-Elements Image Software (v.4.40, Nikon).<br>BD FACSDivaTM (v. 9.0), ProSortTM (v. 1.6) and FlowJo (v. 10.8.1) software were used to analyze the flow cytometry data.<br>Regulatory interactions of over-activated genes in mHTT-resistant clones were analysed with HDnetDB (2017, http://hdnetdb.sysbiolab.eu).<br>Network of HD interacting gene was created by mean of Cytoscape (v3.8.2, http://www.cytoscape.org/).<br>mESCs quantification of nuclear and cytoplasmic intensity was performed by CellProfiler software (v. 4.1.3).<br>A detailed description of all software, including software version and parameters used, for the analysis of RNA-sequencing and Next-Generation sequencing data has been provided in the Methods section.<br>Term enrichment analysis was conducted with Enrichr database (v. 3.0, http://amp.pharm.mssm.edu/Enrichr).<br>Gene set enrichment analysis was performed using GSEA software (v. 4.3.2, http://software.broadinstitute.org/gsea/). |

Jaspar database (9th version, http://jaspar.genereg.net/) and Integrated Genomics Viewer (IGV v. 2.16.0) were used to obtain Mtf1 MRE.
All statistical analyses were carried out in R environment (v. 4.0.0 and v. 4.1.0) with Bioconductor (v. 3.14), Past4.03 or Prism (v. 9.5.0).
The Mtf1 sequence alignment was performed using the Clustal Omega software (v. 1.2.4, https://www.ebi.ac.uk/Tools/msa/clustalo/).

For manuscripts utilizing custom algorithms or software that are central to the research but not yet described in published literature, software must be made available to editors and reviewers. We strongly encourage code deposition in a community repository (e.g. GitHub). See the Nature Portfolio guidelines for submitting code & software for further information.

## Data

Policy information about availability of data

All manuscripts must include a data availability statement. This statement should provide the following information, where applicable:

- Accession codes, unique identifiers, or web links for publicly available datasets
- A description of any restrictions on data availability
- For clinical datasets or third party data, please ensure that the statement adheres to our policy

RNA sequencing data generated during the current study are available via the Gene Expression Omnibus (GEO) repository under the accession numbers GSE166567. All RNA-seq process data, used in Figures 1f-g, 5b-c and in Supplementary Fig. 5a-d, 6d-e are reported in Supplementary Data 1-2-3. Primers and oligonucleotide sequences are present in Supplementary Tables 1,3. Additional data that support the findings of this study, such as analysis pipelines and reagents are available from the corresponding authors upon reasonable request. Source data underlying Fig. 1c-e; 2b-c, e-g; 4b-c, e-h; 5a, e-g; 6c-d,f; 7b-e, g, h, 8d-g and Supplementary data Fig. 1a, c, e-g; 2a, c-d, f; 4a-d; 6a, c; 7c; 8b-c, f-h; 9a, c-d are provided with this paper.

## Human research participants

Policy information about studies involving human research participants and Sex and Gender in Research.

| | |
|---|---|
| Reporting on sex and gender | Not applicable |
| Population characteristics | Not applicable |
| Recruitment | Not applicable |
| Ethics oversight | Not applicable |

Note that full information on the approval of the study protocol must also be provided in the manuscript.

# Field-specific reporting

Please select the one below that is the best fit for your research. If you are not sure, read the appropriate sections before making your selection.

☒ Life sciences ☐ Behavioural & social sciences ☐ Ecological, evolutionary & environmental sciences

For a reference copy of the document with all sections, see nature.com/documents/nr-reporting-summary-flat.pdf

# Life sciences study design

All studies must disclose on these points even when the disclosure is negative.

| | |
|---|---|
| Sample size | Sample size calculation was not performed. Sample size was chosen based on standards in the field (Ying et al. 2008 Nature; Avior et al., 2016) and is indicated in figure legends. |
| Data exclusions | We presented all data obtained, also in the case of negative results. |
| Replication | All key experiments were repeated between two and eight times independently, as indicated in figure legends. Each independent qPCR experiments were performed with three technical replicates. For Next-Generation sequencing experiments we analysed at least three independent biological replicates for each sample. |
| Randomization | Randomization was not relevant for in vitro experiment, however all cell lines or biological samples were analysed or treated in the same manner. Mice were randomly allocated to the AAV-GFP or AAV-Mtf1 groups. |
| Blinding | We applied blinding for RNA-seq and NGS processing and data analysis. Investigators were not blind to cell line identities during all other analyses as knowledge of identity was necessary in order to perform the analysis, but key results were independently replicated by two different operators.<br>Blinding was applied for mice in vivo experiments. |

# Reporting for specific materials, systems and methods

We require information from authors about some types of materials, experimental systems and methods used in many studies. Here, indicate whether each material, system or method listed is relevant to your study. If you are not sure if a list item applies to your research, read the appropriate section before selecting a response.

## Materials & experimental systems

| n/a | Involved in the study |
| --- | --- |
| ☐ | ☒ Antibodies |
| ☐ | ☒ Eukaryotic cell lines |
| ☒ | ☐ Palaeontology and archaeology |
| ☐ | ☒ Animals and other organisms |
| ☒ | ☐ Clinical data |
| ☒ | ☐ Dual use research of concern |

## Methods

| n/a | Involved in the study |
| --- | --- |
| ☒ | ☐ ChIP-seq |
| ☐ | ☒ Flow cytometry |
| ☒ | ☐ MRI-based neuroimaging |

## Antibodies

Antibodies used

Please see Supplementary Table 2 for the list of antibodies, product codes, dilutions used for Western Blot and Immunostaining.
Antibodies for Western Blot: HTT (clone 1HU-4C8, 1:5000, Millipore cat. MAB2166); HTT (EM48 clone, 1:1000 Millipore cat. MAB5374); GAPDH (clone 6C5, 1:2000, Millipore cat. MAB374 ), GFP (rabbit polyclonal antibody, 1:1000, Abcam cat. ab290), α-TUBULIN (clone B-5-1-2, 1:5000, Sigma-Aldrich T5168), β-ACTIN (clone 8H10D10, 1:2000, Cell Signaling Technology cat. 3700).
Antibodies for immunostaining: HTT (EM48 clone, 1:50 Millipore cat. MAB5374); MTF1 (Mouse polyclonal, 1:200, Novus biologicals cat. NBP1-86380); OCT4 (clone C-10, 1:300, Santa Cruz SC-5279); NANOG (clone D73G4, 1:100, Cell Signalling Technology 4903S); PAX6 (clone Poly19013, 1:300, Biolegend 901302); SOX1 (Goat polyclonal, 1:50, R&D AF3369-SP); NESTIN (clone 10C2, 1:500, Sigma-Aldrich MAB5326); OTX2 (Goat polyclonal, 1:100, R&D AF1979); Alexa Fluor 568 (Polyclonal, 1:500, Invitrogen A-10037); Alexa Fluor 647 (Polyclonal, 1:500, Invitrogen A-31573); Alexa Fluor 488 (Polyclonal, 1:500, Invitrogen A-11055).

Validation

All antibodies chosen for this work have been previously used and validated in literature and by manufacturers.
Antibodies were used following manufacturers guidelines and were further validated in our laboratory by using relevant controls or, when possible, independent techniques. See Supplementary Table 5 for all relevant details.
HTT antibody was previously validated in Macdonald, D. et al., PloS one 9(5): e96854 (2014).
HTT (EM48 clone) antibody was previously validated in Wang, C.E., et al., J. Cell Biol., 181, 803–816 (2008).
GAPDH antibody was previously validated in Yao, X. et al., PloS one 10, e0139416 (2015).
GFP antibody was previously validated in Zhou, Y.Q. et al., EMBO Mol Med 12, e10233 (2020).
α-TUBULIN antibody was previously validated in Kalebic, N., et al., Mol Cell Bio 1114–1123 (2013).
β-ACTIN antibody was previously validated in Condeelis, J. et al., Trends Cell Biol 11, 288-93 (2001).
MTF1 antibody was previously validated in Zhang, D. et al., J. Agric. Food Chem 67, 4611-4622 (2019).
OCT4 antibody was previously validated in Szlachcic, W. J. et al., Front Mol Neurosci 8;10:253 (2017).
NANOG antibody was previously validated in Zorzan I. et al., Nat Comm 11,2364 (2020).
PAX6 antibody was previously validated in Ooi J. et al., Cell Rep. 26:2494 (2019).
SOX1 antibody was previously validated in Malankhanova T. et al., J Pers Med 10(4):215 (2020).
NESTIN antibody was previously validated in Ooi J, et al., Cell Rep. 26:2494 (2019).
OTX2 antibody was previously validated in Malankhanova T. et al., J Pers Med 10(4):215 (2020).
Alexa Fluor 568 antibody was previously validated in Zorzan I. et al., Nat Comm 11,2364 (2020).
Alexa Fluor 647 antibody was previously validated in Zorzan I. et al., Nat Comm 11,2364 (2020).
Alexa Fluor 488 antibody was previously validated in Zorzan I. et al., Nat Comm 11,2364 (2020).

## Eukaryotic cell lines

Policy information about cell lines and Sex and Gender in Research

Cell line source(s)

Mouse ES cell lines (Rex1GFP-d2 and E14IVc) kindly provided by Austin Smith's laboratories.
Q15 and Q128 cells were generated by DNA transfection of vectors containing N-terminal of human huntingtin gene, with 128 or 15 CAG repeats respectively (courtesy of Professor Elena Cattaneo).
Stable transgenic mouse ESCs expressing candidates were generated by transfecting cells with PB transposon plasmids (CAG-mcherry, CAG-Mtf1, CAG-Kdm2b, CAG-Kdm5b, CAG-Fbxo34 and CAG-Mtf1+Kdm2b) with PB transposase expression vector pBase.
h-iPSC Q21 and Q109 lines were obtained from Cedars-Sinai.
NPC Q21 and NPC Q109 were differentiated from h-iPSC lines.

Authentication

Every cell line was validated by qPCR and DNA genotyping.

Mycoplasma contamination

Cells were routinely tested for Mycoplasma contamination. All cell lines were Mycoplasma negative.

Commonly misidentified lines
(See ICLAC register)

The cell lines used are not listed in ICLAC.

# Animals and other research organisms

Policy information about studies involving animals; ARRIVE guidelines recommended for reporting animal research, and Sex and Gender in Research

| | |
|---|---|
| Laboratory animals | Breeding pairs of the R6/2 line of transgenic female mice [strain name: B6CBA-tgN (HDexon1) 62Gpb/1J] with ~160±10 (CAG) repeat expansions were purchased from the Jackson Laboratories. Male R6/2 mice (5-6 weeks of age) were crossed with female B6CBA WT mice (5-6 weeks of age) for colony maintenance. Mice used for experiments were treated at 4 weeks of age and analised up to 11 weeks. See Supplementary Table 4 for detailed informations.<br>All Zebrafish experiments were carried out at the Fish Facility in the Department of Biology of the University of Padova. Zebrafish larvae were kept at most three days in Petri dishes with fish water (60 mg of Instant Ocean, cat. no. SS15-10, per litre of distilled water) at neutral pH at 28°C. |
| Wild animals | This study did not involve wild animals. |
| Reporting on sex | Both sex were considered in study design and our findings apply to both sex. For Zebrafish experiments, sex was not determined or collected. For mice experiments sex was determined by visual inspection and collected as follow: 60 female mice, 1 male mouse.<br>ID code and relative sex for each mice is reported in the Supplementary Table 4. |
| Field-collected samples | This study did not involve samples collected from the field. |
| Ethics oversight | All procedures were performed according to protocols approved by the internal institutional animal care and use committee (IACUC) and reported to the Italian Ministry of Health according to the European Commission Council Directive 2010/63/EU and to Italian legislation on animal experimentation (Decreto Legislativo D.Lgs 26/2014).<br>All Zebrafish experiments were carried out according to standard procedures (http://ZFIN.org).<br>All mice experimental procedures were approved by the IRCCS Neuromed Animal Care Review Board ethics committee and by Italian Healthy Department (ISS permit number: n.548/2022-PR). |

Note that full information on the approval of the study protocol must also be provided in the manuscript.

# Flow Cytometry

## Plots

Confirm that:

☒ The axis labels state the marker and fluorochrome used (e.g. CD4-FITC).

☒ The axis scales are clearly visible. Include numbers along axes only for bottom left plot of group (a 'group' is an analysis of identical markers).

☒ All plots are contour plots with outliers or pseudocolor plots.

☒ A numerical value for number of cells or percentage (with statistics) is provided.

## Methodology

| | |
|---|---|
| Sample preparation | Annexin V staining. Live NPCs, transiently transfected with the gene of interest and treated with Rotenone 30 µM for 24 hours, were stained with Annexin V according to the manufacturer's instructions (Ebioscience, cat. 88-8007-72). Cells were washed once in PBS, then once in 1x Binding Buffer (cat. 00-0055). 5x105 cells were resuspended in 200 µL of 1x Binding Buffer and incubated with 5 µL of fluorochrome-conjugated Annexin V (cat. 17-8007) for 10 minutes at room temperature. Cells were then washed in 500 µL of 1x Binding Buffer. Finally, cells were resuspended in 200 µL of 1x Binding Buffer. Flow cytometry analysis was performed using the BIO-RAD S3e Cell Sorter within 1 hour, storing samples at 2-8 °C in the dark. Data were analysed with ProSortTM (v. 1.6) and FlowJo (10.8.1) software.<br><br>Propidium iodide (PI) staining. PI staining was performed on live single mouse ESCs according to the manufacturer's instructions (Ebioscience, cat. 88-8007-72). After washing in PBS, 105 live cells were resuspended in 200 µL of 1x Binding Buffer and 5 µL of PI Staining Solution (cat. 00-6990) were added. Flow cytometry analysis was performed using a BD FACSCantoTM cytometer within 1 hour, storing samples at 2-8 °C in the dark. Data were analysed with BD FACSDivaTM (v. 9.0) and FlowJo (10.8.1) software.<br><br>ROS measurement assay. ROS production was detected by staining single live mouse ESCs and human NPCs cells with 2′,7′-dichlorodihydrofluorescein diacetate (H2DCFDA; Life Technologies, cat. D399), performing the following steps: a) ROS indicator was freshly reconstituted in order to make a concentrated stock solution (10 mM); b) 3-5x105 cells were harvested, c) washed once with 500 µL of PBS and d) resuspend in 300 µL PBS containing the probe to provide a final working concentration of 0.5 µM dye; e) cells were incubated at 37 °C for 10 minutes in the dark; f) after removal of the staining solution, samples were g) washed twice in PBS. Samples were analysed by flow cytometry using a BD FACSDivaTM (v. 9.0), ProSortTM (v. 1.6) and FlowJo (10.8.1) software. |
| Instrument | BD FACSCantoTM II cytometer for mESCs and BioRad Sorter for human NPCs. |
| Software | BD FACSDivaTM (v. 9.0), ProSortTM (v. 1.6) and FlowJo (10.8.1) software were used to collect and analyze the flow cytometry data. |

| | |
|---|---|
| Cell population abundance | Only cell lines were analyzed. |
| Gating strategy | The preliminary FSC/SSC gates were builded on a homogeneous cell population in order to eliminate cell debris. The boundaries between PI and AV positive and negative cells were defined based on cells without staining (blank). The same gating strategy was performed for experiments with DCFDA (Supplementary Fig. 10). |

☒ Tick this box to confirm that a figure exemplifying the gating strategy is provided in the Supplementary Information.
